# Nitrogen Addition Changes the Stoichiometry and Growth Rate of Different Organs in *Pinus tabuliformis* Seedlings

**DOI:** 10.3389/fpls.2017.01922

**Published:** 2017-11-07

**Authors:** Hang Jing, Haoxiang Zhou, Guoliang Wang, Sha Xue, Guobin Liu, Mengcheng Duan

**Affiliations:** ^1^State Key Laboratory of Soil Erosion and Dryland Farming on the Loess Plateau, Institute of Soil and Water Conservation, Northwest A&F University, Yangling, China; ^2^Institute of Soil and Water Conservation, Chinese Academy of Sciences and Ministry of Water Resources, Yangling, China

**Keywords:** nitrogen addition, stoichiometry, growth rate, plant organ, root

## Abstract

**Background:** Nitrogen (N) deposition could influence plant stoichiometry and growth rate and thus alter the structure and function of the ecosystem. However, the mechanism by which N deposition changes the stoichiometry and relative growth rate (RGR) of plant organs, especially roots with different diameters, is unclear.

**Methods:** We created a gradient of N availability (0–22.4 g N m^-2^ year^-1^) for *Pinus tabuliformis* seedlings for 3 years and examined changes in the carbon (C):N:phosphorus (P) ratios and RGRs of the leaves, stems, and roots with four diameter classes (finest roots, <0.5 mm; finer roots, 0.5–1 mm; middle roots, 1–2 mm; and coarse roots, >2 mm).

**Results:** (1) N addition significantly increased the C and N contents of the leaves and whole roots, the C content of the stems, the N:P ratios of the leaves and stems, and the C:P ratio of the whole roots. (2) In the root system, the C:N ratio of the finest roots and the C:P ratios of the finest and finer roots significantly changed with N addition. The N:P ratios of the finest, finer, and middle roots significantly increased with increasing amount of N added. The stoichiometric responses of the roots were more sensitive to N addition than those of the other organs (3) The RGR of all the organs significantly increased at low N addition levels (2.8–11.2 g N m^-2^ year^-1^) but decreased at high N addition levels (22.4 g N m^-2^ year^-1^). (4) The RGRs of the whole seedlings and leaves were not significantly correlated with their N:P ratios at low and high N addition levels. By contrast, the RGRs of the stems and roots showed a significantly positive correlation with their own N:P ratio only at low N addition level.

**Conclusion:** Addition of N affected plant growth by altering the contents of C and N; the ratios of C, N, and P; and the RGRs of the organs. RGR is correlated with the N:P ratios of the stems and roots at low N addition level but not at high N addition level. This finding is inconsistent with the growth rate hypothesis.

## Introduction

Increasing global atmospheric N deposition could influence the stoichiometry of plant aboveground organs and roots and thus alter the physiological activity and growth rates (Niklas et al., 2005). Such changes could influence the composition, structure, and function of the plant ecosystem (Liu et al., 2011). However, the mechanism by which N deposition changes the stoichiometry and growth rate of plant organs, especially roots with different diameters or orders, is unclear; thus, the understanding of plant growth processes and mechanism is limited, and the development of a plant growth model is hindered (Findenegg, 1990; Yu et al., 2012).

Previous studies on the relationship between plant stoichiometry and environmental changes focused only on plant aboveground organs or leaf stoichiometry (Güsewell, 2004; Cui et al., 2010). For example, Cui et al. (2010) studied the stoichiometry of nine major species in temperate grassland and found that N addition significantly increases the N and P contents and decreases the C:N and C:P ratios of leaves. Meanwhile, Mo et al. (2015) indicated that N addition significantly increases the P content but non-significantly affects the N content and N:P ratio of plant stems. Recent studies have found that the stoichiometry of roots is more sensitive to environmental changes than that of the leaves (Minden and Kleyer, 2014; Schreeg et al., 2014). However, the effects of soil available N on root stoichiometry remain unclear (Mo et al., 2015). Hu et al. (2007) found that N addition does not significantly affect the N and P contents and the N:P ratio of *Ophiopogon japonicus* roots. By contrast, studies on seven tree species in tropical forests reported that N addition increased the N content and N:P ratio of fine roots (Mo et al., 2015). These contradicting results may be due to the different plant species, N addition levels, and soil initial N contents used (Li et al., 2015). However, recent studies have shown that the varied conclusions can be attributed to the different responses of the stoichiometry of fine roots with different diameters to changes in soil available N.

An increasing number of studies showed that N addition induced varied effects on the stoichiometry of roots with different orders and diameters. For example, Guo et al. (2004) found that N addition significantly increased the N contents of the first five root orders of longleaf pine. However, Pregitzer et al. (2002) applied N fertilization to nine tree species and observed that the N content of fine roots of the first three orders increased significantly in three tree species; however, those of the six other species showed no significant response. Moreover, stoichiometric changes of the root system often altered root physiological activities. For example, increasing root N content may increase the respiration rate of fine roots instead of the coarse roots, decrease root longevity, and increase root potential decomposition rate (Guo et al., 2004; Chen et al., 2016). Systematic studies on the effects of soil available N on the stoichiometry of roots with different diameters or orders may be helpful to elucidate the mechanism underlying root growth. However, most studies on root stoichiometry mainly focused on N content and disregarded C and P contents and C:N:P ratios (Pregitzer et al., 2002). Only a few studies analyzed the stoichiometric changes of fine roots based on the root diameter or root orders. Moreover, most studies added 5–15 g N m^-2^ year^-1^, which is insufficient to change soil N content from limitation to saturation for plant growth in most ecosystems (Lu et al., 2009). Thus, we hypothesize that the stoichiometry of C, N, and P varies in plant leaves, stems, and roots with different diameters and that N addition causes different effects on the stoichiometry of plant organs. We also hypothesize that the stoichiometry of the roots is more sensitive to N addition than that of the leaves because roots absorb N from soil. Based on these hypotheses, we try to find the potential mechanism underlying the coupled changes between plant stoichiometry and physiological function at the plant organ level induced by soil N content changes.

The growth rate hypothesis (GRH) indicates that fast-growing plants have low N:P and C:P ratios because of the differential allocation to P-rich ribosomal RNA, which results in a negative relation between growth rate and N:P ratio (Sterner and Elser, 2002). Many studies have validated the GRH in microbes, zooplanktons, arthropods, and insects (Main et al., 1997; Elser et al., 2003; Makino et al., 2003; Acharya et al., 2004). However, the test of GRH to higher plants showed inconsistent results. Some studies support the GRH (Vrede et al., 2004; Niklas and Cobb, 2005; Lovelock et al., 2007). However, a few scholars reported that higher plants can store extra nutrients and thus change the relationship between growth rate and N:P ratio. Thereby, the applicability of GRH to higher plants should be modified (Ågren, 2004, 2008; Cernusak et al., 2010; Yu et al., 2012). The allometric growth of different organs of higher plants may be another reason for the inconsistent results. The functional balance hypothesis suggests that increasing soil available N content increases the aboveground growth rate of plants and relatively decreases the belowground growth rate (Pan et al., 2005; Zhang et al., 2009). Nevertheless, the effect of soil available N on the N:P ratio of different plant organs remains controversial. For example, increasing the soil available N content increased (Cui et al., 2010), decreased (Lü et al., 2012), or did not change (Hu et al., 2007) the N:P ratio of plant organs. Hence, growth rate and N:P ratio may exhibit significant positive correlations, significant negative correlations, irrelevant, or other relationships of plant organs. To the best of our knowledge, few studies have investigated the effects of N addition on the relationship between growth rate and N:P ratio of different plant organs. Moreover, the relationships between plant relative growth rate (RGR) and N:P ratio can change under different soil nutrient conditions (Yu et al., 2012). Increasing the level of N enrichment from deposition alters the N cycle in some ecosystems, thereby shifting the nutrient conditions from N limitation to saturation (Holland et al., 1999). Therefore, the relationship between plant RGR and N:P ratio would be changed. These changes regulate the growth rate, stoichiometry of individual plants, structure, and biogeochemical cycle at the ecosystem level (Aber et al., 1998). The effects of increasing soil available N content on the growth rate, stoichiometric ratio, and relationship between growth rate and N:P ratio of plant organs must be determined to clarify the mechanism underlying N deposition, affecting plant growth (Liu et al., 2011; Wang et al., 2011). Thus, we propose the second hypothesis that N addition alters the plant growth and growth rate related to the N:P ratio of different plant organs; however, these relationships change at different N addition levels.

In this study, we tested the two hypotheses by determining the growth rates and stoichiometry of leaves, stems, and roots with four diameters (<0.5, 0.5–1, 1–2 and >2 mm) of *Pinus tabuliformis* seedlings. We created N availability levels from 2.8 g N m^-2^ year^-1^ to 22.4 g N m^-2^ year^-1^ to determine the possible critical values that promote or limit plant growth and alter the relationship between growth rate and N:P ratio from coupling to decoupling.

## Materials and Methods

### Study Area

The experiment was conducted at the Institute of Soil and Water Conservation in Yangling, Shaanxi Province, China (107°38′E, 33°40′N). The region has a classic temperate continental climate with a mean annual precipitation of 674.3 mm, a mean annual temperature of 13.2°C, a sunshine period of 1993.7 h, and a frost-free period of 225 days.

### Experimental Design

Gray forest soil (Gray Luvisols, FAO soil classification) was collected from Yichuan in Shaanxi Province, China. The soil was sieved through a 2 mm mesh and then transferred to 175 pots. Each pot weighted 18 kg (depth of 35 cm, diameter of 40 cm). One-year-old *P. tabuliformis* seedlings with 3 cm height and similar growth characteristics were transplanted to pots in March 2011 and were grown for 3 years in a free air field setting under five levels of N addition (0–22.4 g N m^-2^ year^-1^) in the form of urea. Urea (Fumin Agriculture Product Company, Xi’an, China) was dissolved in 10 mL of distilled water and evenly added to the pot during a rain fall event in March of each year from 2011 to 2014. The levels of N addition for the five fertilization treatments were 0 (control, CK), 2.8, 5.6, 11.2, and 22.4 g N m^-2^ year^-1^ (0, 0.57, 1.15, 2.3, and 4.6 g urea pot^-1^ year^-1^). The five N addition treatments were observed, and 35 pots were used for each N treatment; this study had a total of 175 pots.

### Sampling and Analysis

Samples were harvested at two growth stages. In stage I, 1-year-old *P. tabuliformis* seedlings with 3 cm height and similar characteristics were separated into three parts: leaves, stems, and roots to measure initial biomass in March 2011. In stage II, mean height (m) and basal diameter (cm) of *P. tabuliformis* seedlings were measured from each treatment at the growing season in July 2014. Organs were collected from four seedlings with mean height and basal diameter for each treatment (four seedlings can represent the properties of treatment and meet the requirements of mathematical analysis). Each individual was separated into six parts: leaves, stems, and four diameter root classes (finest roots: 0–0.5 mm, finer roots: 0.5–1 mm, middle roots: 1–2 mm, and coarse roots: >2 mm). The six parts were immediately weighed when samples were fresh. The leaves, stems, and roots were rinsed with deionized water before drying and grinding to a fine powder. All plant organs were oven-dried at 65 °C for 2 days to a constant weight, weighed, finely ground in a ball mill, and then stored until chemical analysis. The organs’ total C content (mg g^-1^) was determined through H_2_SO_4_–K_2_Cr_2_O_7_ oxidation (Lu, 2000). Total P content (mg g^-1^) was determined by persulfate oxidation followed by colorimetric analysis (Lu, 2000). The total N content (mg g^-1^) was determined colorimetrically by Kjeldahl acid digestion with an Alpkem auto-analyzer (Kjektec System 1026 distilling unit, Sweden) after extraction with sulfuric acid (Bremner and Mulvaney, 1982). The heterostasis of plant organs can be expressed as relative standard deviation (RSD %), which is an index of plant organ’s stoichiometry sensitivity response to N addition. RSD = Standard deviation/Arithmetic mean × 100. RGR was calculated using the following equation:

$$\text{RGR=}\frac{{\text{lnM}}_{\text{t2}}{\text{-lnM}}_{\text{t1}}}{\Delta \text{t}}$$

*M*_t1_: Initial organ (leaves, stems, and roots) biomass at stage I (g). *M*_t2_: Final organ [leaves, stems, and whole roots (with four root diameters)] biomass at stage II (mg). Δ*t*: Days from stages I–II.

### Statistical Analyses

Differences in element content and stoichiometric ratio between any two N addition treatments were tested using one-way ANOVA on SPSS 20.0 statistical software package (SPSS, Inc., United States). Linear regression analyses were used to test the stoichiometric relationships and element contents between leaves and four-diameter root classes, as well as the relationships between RGR and N:P ratio.

## Results

### Effects of N Addition on Element Content of Seedling Organs

**FIGURE 1:**
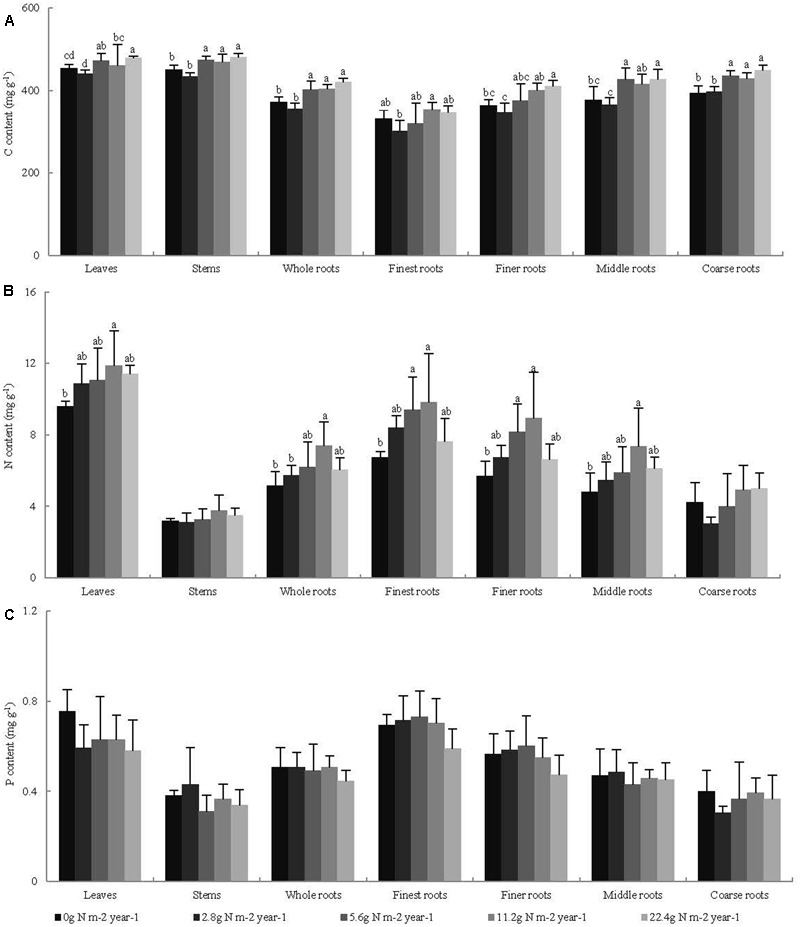
Effects of N addition on the element content of seedling organs, **(A)** C content, **(B)** N content, and **(C)** P content, (*n* = 4). Different letters indicate significant difference of C, N, and P contents (mg g^-1^) among different N addition gradients (*P* < 0.05). The diameter classes for finest, finer, middle, and coarse roots are 0–0.5, 0.5–1, 1–2, and >2 mm, respectively.

Across all seedling organs, the leaves and stems had higher C content than the whole roots. The C content of the roots increased with increasing root diameter. The leaves had higher N and P contents than the other organs. The N and P contents of the roots decreased with increasing root diameter (**Table 1**). In general, the C and N contents of the leaves and whole roots and the C content of the stems significantly increased with N addition, whereas the P content of the plant organs did not significantly change with N addition (**Figure 1**). Across all seedling organs, the RSD of elemental contents was highest in roots, except the RSD of P content (**Table 1**). These results indicate that the responses of C and N contents of roots are more sensitive to N addition than those of leaves and stems.

**Table 1:** Mean (mg g^-1^) and relative standard deviation (RSD, %) of element contents across N addition gradient.

	*C*	*N*	*P*
Leaves	461.22 (3.58)^a^	10.96 (12.77)^a^	0.638 (20.85)^a^
Stems	461.94 (4.53)^a^	3.37 (16.61)^e^	0.365 (24.93)^d^
Whole roots	390.82 (6.94)^cd^	6.10 (19.18)^d^	0.491 (15.07)^c^
Finest roots	331.27 (9.55)^e^	8.40 (22.02)^b^	0.687 (14.41)^a^
Finer roots	379.17 (8.60)^d^	7.23 (24.48)^c^	0.555 (17.66)^b^
Middle roots	402.45 (8.68)^c^	5.93 (24.96)^d^	0.459 (17.86)^c^
Coarse roots	420.93 (6.07)^b^	4.24 (30.66)^e^	0.366 (26.50)^d^

*Different letters indicate significant difference for element contents among the different organs (*p* < 0.05, *n* = 20). The diameter classes for finest, finer, middle, and coarse roots are 0–0.5, 0.5–1, 1–2, and >2 mm, respectively.*

### Effects of N Addition on Stoichiometric Ratio of Seedling Organs

**FIGURE 2:**
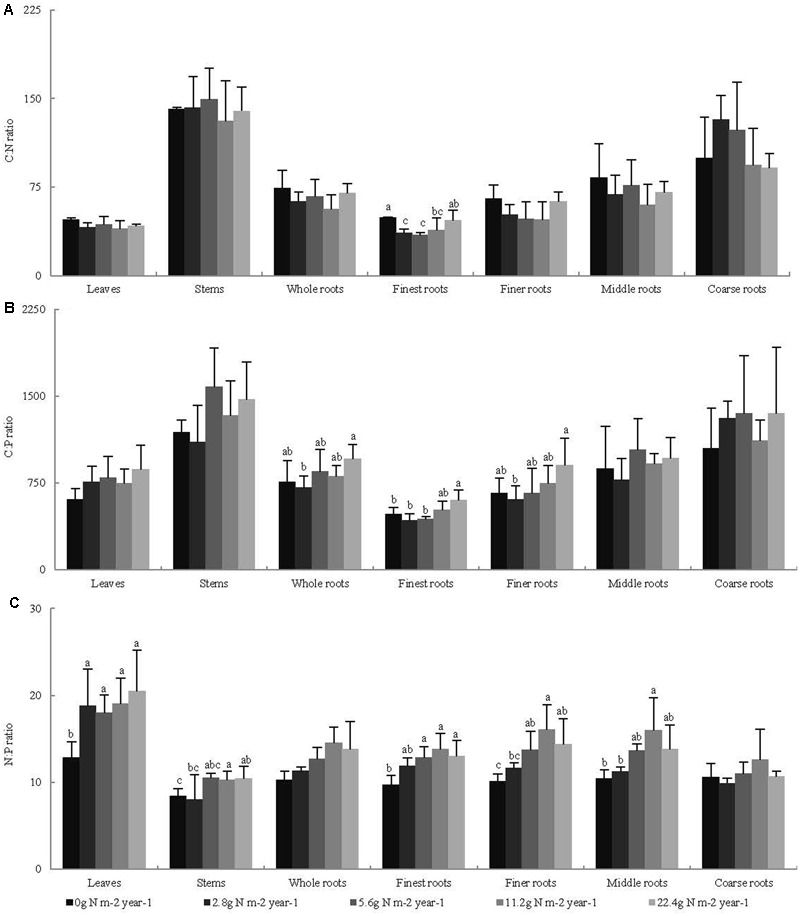
Effects of N addition on stoichiometric ratio of seedling organs, **(A)** C:N, **(B)** C:P, and **(C)** N:P, (*n* = 4). Different letters indicate significant difference of stoichiometric ratio among different N addition gradients (*P* < 0.05). The diameter classes for finest, finer, middle, and coarse roots are 0–0.5, 0.5–1, 1–2, and >2 mm, respectively.

Across all seedling organs, the leaves had the highest N:P ratio, and the stems had the highest C:N and C:P ratios among all organs tested. The C:N and C:P ratios of the roots increased with increasing root diameter (**Table 2**). The N:P ratios of the leaves and stems and the C:P ratios of the whole roots significantly increased with N addition (**Figure 2**). In the root system, the C:N ratio of the finest roots and the C:P ratios of the finest and finer roots significantly changed with N addition. The N:P ratios of the finest, finer, and middle roots significantly increased with N addition. The RSD of the C:N ratio of roots was higher than those of leaves and stems. Meanwhile, the RSD of the C:P and N:P ratios varied among seedling organs (**Table 2**).

**Table 2:** Mean and relative standard deviation (RSD, %) of stoichiometric ratios across N addition gradient.

	*C:N*	*C:P*	*N:P*
Leaves	42.66 (11.88)^e^	754.12 (21.48)^c^	17.84 (22.53)^a^
Stems	140.59 (15.95)^a^	1334.24 (23.61)^a^	9.56 (18.31)^c^
Whole roots	66.09 (18.69)^cd^	815.91 (19.05)^bc^	12.52 (18.21)^b^
Finest roots	40.91 (20.53)^e^	491.82 (17.43)^d^	12.25 (15.59)^b^
Finer roots	55.10 (23.52)^d^	714.31 (26.68)^c^	13.19 (21.61)^b^
Middle roots	71.73 (26.78)^c^	913.16 (24.74)^b^	13.03 (21.57)^b^
Coarse roots	108.02 (28.96)^b^	1234.77 (29.72)^a^	11.98 (38.73)^b^

*Different letters indicate significant difference for stoichiometry ratio among different organs (*p* < 0.05, *n* = 20). The diameter classes for finest, finer, middle, and coarse roots are 0–0.5, 0.5–1, 1–2, and >2 mm, respectively.*

### Linear Regression Analysis of Stoichiometric Ratio between the Leaves and the Root System

In most cases the element contents and the C:N:P ratios of the leaves correlated significantly with those of the root system, except for the five relationships that were non-significant (**Table 3**). Any changes in the stoichiometric ratio or element content of the leaves across the N addition treatments were accompanied by larger changes of the root system except for three relationships (**Table 3**, slope <1).

**Table 3:** Correlation coefficient and slope of the linear regression (in the parentheses) for the linear regression analysis of element content (upper) and stoichiometry ratio (lower) between the leaves and roots with four diameters.

*Y*	*X*	*C*	*N*	*P*
Leaves	Finest roots	NS	(0.533^∗^, 0.404)	(0.479^∗^, 0.640)
Leaves	Finer roots	(0.620^∗^, 0.314)	(0.595^∗^, 0.470)	(0.546^∗^, 0.736)
Leaves	Middle roots	(0.781^∗^, 0.370)	(0.645^∗^, 0.609)	NS
Leaves	Coarse roots	(0.635^∗^, 0.411)	(0.469^∗^, 0.503)	(0.717^∗^, 0.982)
Leaves	Whole roots	(0.774^∗^, 0.471)	(0.744^∗^, 0.888)	(0.716^∗^, 1.289)

***Y***	***X***	***C:P***	***N:P***	***C:N***

Leaves	Finest roots	NS	(0.807^∗^, 1.694)	(0.418^∗^, 0.252)
Leaves	Finer roots	(0.550^∗^, 0.468)	(0.715^∗^,0.007)	(0.628^∗^, 0.246)
Leaves	Middle roots	(0.462^∗^, 0.331)	(0.498^∗^, 0.712)	(0.633^∗^, 0.167)
Leaves	Coarse roots	(0.705^∗^, 0.311)	NS	NS
Leaves	Whole roots	(0.717^∗^, 0.747)	(0.635^∗^, 1.120)	(0.712^∗^, 0.292)

*NS indicates no significant relationship, and ^∗^ indicates a significant relationship at *p* < 0.05 (*n* = 20). The diameter classes for finest, finer, middle, and coarse roots are 0–0.5, 0.5–1, 1–2, and >2 mm, respectively.*

### Effects of N Addition on RGR of Seedling Organs

Relative growth rate varied among different seedling organs. Compared with the other organs, the stems had higher RGR whereas the roots had lower RGR (**Table 4**). The RGR of the whole plants and organs significantly increased with N addition at 0–11.2 g N m^-2^ year^-1^ treatments and then significantly decreased at 22.4 g N m^-2^ year^-1^ treatments compared with the control.

**Table 4:** Effects of N addition on relative growth rate of whole seedling and organs.

Treatment (g N m^-2^ year^-1^)	Whole plant relative growth rate	Leaves relative growth rate	Stems relative growth rate	Whole roots relative growth rate	Whole plant biomass (g seedling^-1^)
	(mg g^-1^d^-1^)	(mg g^-1^d^-1^)	(mg g^-1^d^-1^)	(mg g^-1^d^-1^)	
CK (0)	1.596 ± 0.056^c^	1.803 ± 0.086^ab^	1.639 ± 0.048^d^	1.090 ± 0.046^b^	365.41 ± 29.04^c^
N1 (2.8)	1.746 ± 0.058^b^	1.809 ± 0.080^ab^	2.077 ± 0.049^b^	1.107 ± 0.085^b^	504.38 ± 40.77^b^
N2 (5.6)	1.745 ± 0.081^b^	1.889 ± 0.099^a^	1.988 ± 0.084^c^	1.073 ± 0.051^b^	558.64 ± 63.23^b^
N3 (11.2)	2.049 ± 0.048^a^	1.784 ± 0.068^ab^	2.398 ± 0.040^a^	2.069 ± 0.039^a^	837.44 ± 57.06^a^
N4 (22.4)	1.603 ± 0.033^c^	1.731 ± 0.032^b^	1.986 ± 0.048^c^	0.949 ± 0.010^c^	316.26 ± 14.50^c^

*Different letters indicate significant difference for a given index among different N additions (*p* < 0.05). CK, N1, N2, N3, and N4 are 0, 2.8, 5.6, 11.2, and 22.4 g N^-*2*^ year^-*1*^, respectively.*

### Relationships between RGR and N:P Ratio

**FIGURE 3:**
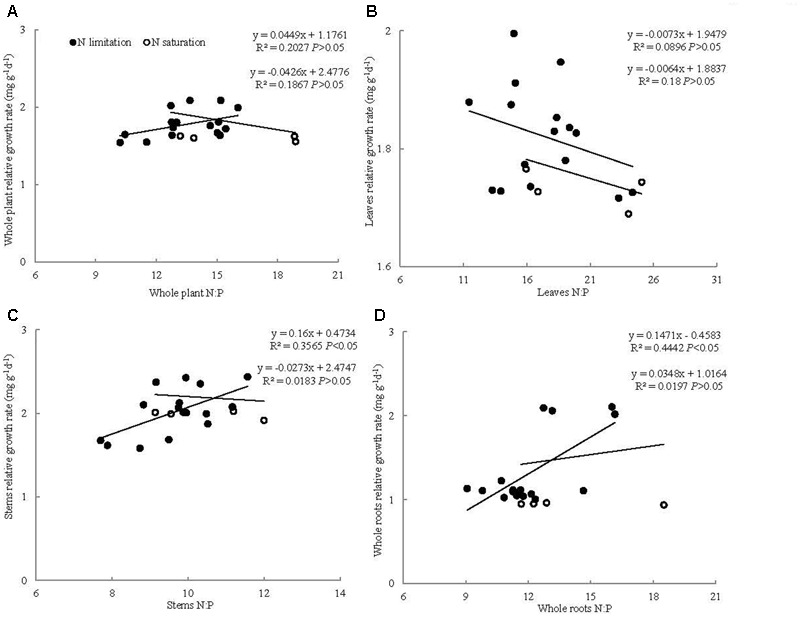
Relationships between N:P ratio and relative growth rate (mg g^-1^ d^-1^) for **(A)** whole plant, **(B)** leaves, **(C)** stems, and **(D)** roots in *P. tabuliformis* seedling.

The relationships between RGR and N:P ratio varied under different N addition ranges. The RGR of the whole seedlings and leaves showed no significant correlation with their N:P ratios at 0–11.2 and 11.2–22.4 g N m^-2^ year^-1^ treatments. The RGRs of the stems and whole roots had significantly positive correlation with their own N:P ratios at 0–11.2 g N m^-2^ year^-1^ but not at 11.2–22.4 g N m^-2^ year^-1^ treatments (**Figure 3**).

## Discussion

### Effects of N Addition on the Stoichiometry of Seedling Organs

In this study, the stoichiometric ratios varied among the different plant organs. N addition significantly changed the stoichiometric ratios and element contents (except P content) of the plant organs. In addition, stoichiometry of root was more sensitive to the changes of soil available N content compared with those of the other organs. These results partly support our first hypothesis.

Many studies found that the stoichiometry of different organs varies (Kleyer and Minden, 2015). For example, some studies showed that the N and P contents and N:P ratio of the leaves were higher than those of the other organs, whereas the C content and C:N and C:P ratios of the stems were the highest among all organs (Luo et al., 2006; Li et al., 2013). These findings are consistent with our results. In addition, the C content and C:N and C:P ratios increased, but the N and P contents decreased with increasing root diameter in our research, which is consistent with the results of Ma et al. (2015). Many studies showed that the stoichiometric ratios of the roots with different diameters was coupled with their physiological functions. For example, the finest, finer, and middle roots are the main organs for water and nutrient uptake, with high respiration and turnover rates, whereas the coarse roots are the main organs for water and nutrient transport, with low respiration and turnover rates (Wang et al., 2013). Our results also showed that the N:P ratio did not decrease with increasing root diameter, which is inconsistent with the results of Ma et al. (2015). These inconsistencies may result due to the different soil nutrients, regional climates, plant species, or study methods used (Pregitzer et al., 2002). The different stoichiometry of the roots may be consistent with their physiological functions of roots with different diameters or orders. For example, many studies found that finer roots with high N contents exhibit higher respiration rates and mycorrhizal colonization rates, as well as an active uptake of nutrients compared with coarser roots (Sun et al., 2012; Wang et al., 2013). Many studies have reported that the morphological, anatomical, and physiological characteristics of the root system vary hierarchically between root orders and diameters (Pregitzer et al., 2002; Guo et al., 2008; Yuan et al., 2011; Sun et al., 2012). Such variation also applies to the stoichiometric characteristic of the root system.

Stoichiometry mainly reflects the ability of plants to utilize C, N, and P, which are susceptible to environmental changes (Sardans et al., 2012). In this study, N addition significantly increased the C and N contents of the plant leaves and whole roots and the C content of the stems; however, N addition non-significantly changed the P content of the plant organs. These phenomena, which are consistent with previous studies, caused changes in the C:P, N:P, and C:N ratios of plant organs (Zhang et al., 2004; An et al., 2011; Bin et al., 2014; Shi et al., 2015). N:P ratio is widely accepted as an efficient indicator of plant nutrient condition in response to environmental changes (Griffiths et al., 2012). In this study, N addition significantly increased the N:P ratios of the seedling leaves and stems but exerted no significant effects on the whole roots; this result is consistent with the result of a previous research (Zhang et al., 2016). Other related studies have also found that N addition increased (Chen et al., 2015) or decreased (Wei et al., 2013) the N:P ratio of plant leaves, stems, and roots simultaneously or individually (Zhan et al., 2016). Therefore, the effect of N addition on the stoichiometry of plants generally varies depending on soil initial N content, climate, vegetation type, and other factors (Lü et al., 2013; Mayor et al., 2014).

The effects of N addition on plant stoichiometry are different not only in different organs but also in the same organ. In this study, N addition significantly changed the C:N ratio of the finest roots and the C:P ratios of the finest and finer roots. Moreover, N addition significantly increased the N:P ratios of the finest, finer, and middle roots. In general, the changes in the stoichiometric ratio were greater in finer roots than in coarse roots. The reasons for these changes may be as follows: N addition would change the morphological and physiological characteristics of the root system; this change increases the number, length, production, turnover, and biomass of finer roots but does not affect coarse roots (Wang et al., 2017). These hierarchical changes were formed by the responses of stoichiometry to N addition. The stoichiometric changes of the different-diameter roots would also affect the physiological functions, such as enhance the water and nutrient uptake, and increase the respiration and turnover rates of fine roots but not of coarse roots. These changes further decrease the C allocation of the belowground (Wang and Liu, 2014), thereby altering plant growth strategies (Wang et al., 2013) and C cycling in the ecosystem (Pregitzer et al., 2002). Chen et al. (2016) found that N addition exerts the same effects on five-order roots, which contradicts with our results. In addition, recent studies have shown that N addition increased the N content of fine roots but exerted no significant effect on coarse roots (Yu et al., 2009). These inconsistent results may be related to plant species, soil initial N content, N addition level, and duration.

Homeostasis is the ability to maintain stable nutrient content of plant organs despite fluctuation in environmental resources (Cannon, 1929; Bradshaw et al., 2012). Plant organs vary in homeostatic ability; thus, different organs have diverse responses to environmental changes. Plant organ stoichiometry can be an indicator of environmental change beyond what homeostasis controls (Schreeg et al., 2014). Two results in the present study indicated that the stoichiometry of the roots is a more responsive indicator to N addition than those of the other organs. First, linear regression analysis showed that most stoichiometric relationships between the leaves and the roots were significant (except five relationships, **Table 3**). Furthermore, the slopes of the linear regression between the leaves (*Y*) and the roots (*X*) were always less than 1 (except three slopes), indicating that any change in the element content or stoichiometric ratio of the leaves in response to N addition was accompanied by larger changes of the roots. Second, heterostasis can be expressed as RSD (Minden and Kleyer, 2014), and the RSD of the N and C contents and C:N ratio of the roots was higher than those of the other organs across all N addition treatments. This finding indicates that the variation in C and N contents and C:N ratio of the roots were greater than those of the other organs in response to N addition. These two lines of evidence demonstrate that the stoichiometry of the roots is a more responsive indicator of N addition than those of other organs. Our results are consistent with those of previous studies (Li et al., 2014; Minden and Kleyer, 2014; Schreeg et al., 2014). An explanation is that the leaves have higher N and P contents than other organs that are generally metabolic and reproductive. Thus, the leaves need a higher level of homeostasis than the roots to ensure key plant physiological activities when soil N content changes (Li et al., 2014). These findings indicate that plant C gain needs a more stable and optimal stoichiometric ratio than the absorbing function (Minden and Kleyer, 2014). This stoichiometric mechanism is favorable in understanding plant survival strategies. The stoichiometry of the roots, as the main organ of nutrient absorption, is more sensitive to N addition than those of the other organs. However, the sensitivity of root stoichiometry to N addition did not vary with root diameter. Further studies are needed in the future to elucidate the underlying mechanism.

### Relationships between RGR and N:P Ratio

The RGR differed among seedling organs. N addition significantly increased the RGRs at 0–11.2 g N m^-2^ year^-1^ treatments but significantly decreased the RGRs at 22.4 g N m^-2^ year^-1^ treatments. Previous studies found that the research region is an N-limited region (Wang et al., 2013). Thus, N addition facilitated seedling growth at 0–11.2 g N m^-2^ year^-1^. However, N addition at 22.4 g N m^-2^ year^-1^ decreased plant growth rates, possibly indicating that the critical value for N saturation is between 11.2 and 22.4 g N m^-2^ year^-1^. In addition, the relationships between RGR and N:P ratio varied among the different seedling organs, and these relationships were determined by N addition level. These results partly supported our second hypothesis.

The N:P ratio of whole seedlings showed no significant correlation with its own RGR, which does not support the RGH at the whole seedling level. This result can be attributed to the phenomenon that N addition exerted no significant influence on N:P ratios but increased seedling growth at low N addition and restrained growth at high N addition. Our results are consistent with the findings of Matzek and Vitousek (2009), who suggested that the relationships between N:P ratio and RGR vary. Meanwhile, seedling stem and whole root N:P ratios had a positive relationship with their own RGR at low N addition levels. These results are analogous with those of Yu et al. (2012), who found that plant aboveground and belowground RGRs are positively correlated with N:P ratio under N addition treatment. In N-limited regions, whole root N:P ratios increased with N addition. Meanwhile, N addition significantly increased RGR, which resulted in the positive relationship between the RGR and N:P ratio of whole roots. Similarly, N addition significantly increased the RGR and N:P ratio of the stems under N-limited growth condition, indicating a positive relationship. Conversely, under N-saturated condition, the relationships between the RGR and N:P ratio of the stems and whole roots were not significant. This result may be due to the phenomenon that N addition significantly decreased RGR but non-significantly decreased the N:P ratio of the stems and whole roots. Thus, the applicability of GRH to woody plant organs may be related to N addition level (Yan et al., 2015). In addition, the relationship between RGR and N:P ratio of the leaves was non-significant at any N addition level, given that the RGR and N:P ratio of the leaves were non-significantly different among the different N addition levels. These results indicate that GRH is unsupported in plant organs. Peng et al. (2011) found that the relationship between RGR and N:P ratio differs between shrub and herbaceous species (significant or non-significant relationships). Our results proved that the relationship between RGR and N:P ratio of woody whole plants was different as well. Not only the relationship between RGR and N:P ratio of whole plants was different among species but also among plant organs. These results indicated that the relationship between RGR and N:P ratio varied among woody plant organs, which may lead to higher plant GRH controversies. Moreover, the applicability of GRH to higher plants might be related to N addition ranges.

## Conclusion

The responses of stoichiometry to N addition varied among *P. tabuliformis* seedling organs, especially among roots with different diameters. Furthermore, the stoichiometry of the roots was more responsive to N addition than those of the other organs. Seedling growth was facilitated at low N addition levels (0–11.2 g N m^-2^ year^-1^) but was restrained at high N addition levels (11.2–22.4 g N m^-2^ year^-1^). A positive correlation between RGR of the stems and roots with their own N:P ratio was observed at low N addition levels. These findings are inconsistent with GRH. At high N addition level, however, the correlations were decoupling. Our conclusion are based on the seedling plant, and the studies on mature tree are needed in the future.

## Author Contributions

GW, GL, and SX conceived and designed the study. HZ and MD performed the experiments. HJ wrote the paper. GW, HJ, and GL reviewed and edited the manuscript. All authors read and approved the manuscript.

## Conflict of Interest Statement

The authors declare that the research was conducted in the absence of any commercial or financial relationships that could be construed as a potential conflict of interest.
